# Treatment of hip/knee osteoarthritis in Dutch general practice and physical therapy practice: an observational study

**DOI:** 10.1186/s12875-015-0295-9

**Published:** 2015-06-27

**Authors:** Di-Janne JA Barten, llse CS Swinkels, Sara A Dorsman, Joost Dekker, Cindy Veenhof, Dinny H de Bakker

**Affiliations:** NIVEL (Netherlands Institute for Health Services Research), PO Box 1568, 3500 BN Utrecht, The Netherlands; Department of Rehabilitation Medicine, VU University Medical Center, Amsterdam, The Netherlands; Department of Rehabilitation, Nursing Science and Sport, University Medical Center Utrecht, Utrecht, The Netherlands; Tilburg University, Scientific Centre for Transformation in Care and Welfare (TRANZO), Tilburg, The Netherlands

**Keywords:** Osteoarthritis, Primary health care, General practice, Physical therapy specialty, Community health services, Referral and consultation

## Abstract

**Background:**

A multidisciplinary, guideline-based Stepped-Care-Strategy (SCS), has recently been developed to improve the management of hip and knee osteoarthritis (OA). To date, it is unknown to what extent current Dutch OA care is consistent with the SCS, both with respect to the content of care as well as the sequence of care. Furthermore, there is a lack of clarity regarding the role of different health care providers in the performance of OA care according to the SCS. Therefore, the main purpose of this study is to describe the content of primary care in patients with hip/knee OA, including the compliance to the SCS and taking into account the introduction of patient self-referral to physical therapy.

**Methods:**

Data were used from NIVEL Primary Care Database. In total, 12.118 patients with hip/knee OA who visited their GP or physical therapist were selected. Descriptive statistics were used to compare the content of care in GP-referred and self-referred patients to physical therapy.

**Results:**

Content of care performed by GPs mostly concerned consultations, followed by NSAID prescriptions and referrals to secondary care. Both prescriptions of acetaminophen and referrals to physical therapy respectively dietary therapy were rarely mentioned. Nevertheless, still 65% of the patients in physical therapy practice were referred by their GP. Compared to GP-referred patients, self-referred patients more often presented recurrent complaints and were treated less often by activity-related exercise therapy. Education was rarely registered as singular intervention, neither in GP-referred nor in self-referred patients.

**Conclusion:**

In accordance with the SCS, less advanced interventions are more often applied than more advanced interventions. To optimize the adherence to the SCS, GPs could reconsider the frequent use of NSAIDs instead of analgesics and the low referral rate to allied health care. Self-referral to physical therapy partially distorts both the low referral rate in general practice and the low application rate of education as singular intervention in physical therapy practice. Further research is recommended to evaluate the effects of task-shifting in OA care, taking into account the content of the SCS.

**Electronic supplementary material:**

The online version of this article (doi:10.1186/s12875-015-0295-9) contains supplementary material, which is available to authorized users.

## Background

Osteoarthritis (OA) is one of the most common disorders of the musculoskeletal system [1]. As a consequence of the aging process, a large increase of the OA population is expected over the next decades [2]. Considering OA as the major cause of musculoskeletal pain and disability in the elderly, a large increase of demand for care could be expected as well [3]. To cope with this demand, it is important to manage OA in an effective and efficient way. Over the last decades, more than 50 modalities of non-pharmacological, pharmacological and surgical interventions for hip and knee OA have been described in medical literature and integrated in (inter)national, monodisciplinary and interdisciplinary clinical guidelines [3–5]. Recently, Smink et al. developed a multidisciplinary, guideline-based Stepped-Care-Strategy (SCS), known as BART, i.e. Beating Osteoarthritis, to improve the management of hip and knee OA [6]. In addition to current clinical guidelines that recommend appropriate non-surgical treatment modalities, the SCS focuses on the optimal order in which to employ them. It recommends offering all modalities in the previous steps before turning to more advanced modalities in the subsequent steps. According to the SCS, treatment of hip/knee OA starts in primary care with stimulating patients’ self-care by emphasizing the usefulness of an adequate dose of acetaminophen and by educating patients about OA and their lifestyle (step 1). Additionally, the use of glucosaminesulphate could be considered for a trial period of three months. In case of persisting complaints, which is identified during an evaluation visit at the general practitioner (GP), (topical) non-steriodal anti-inflammatory drugs (NSAIDs) or tramadol are applied, supplemented by prescribing exercise therapy and, in case of overweight, dietary therapy to diminish the impairments and limitations due to OA (step 2). A referral to secondary care, TENS and intra-articular corticosteroid injections could be applied as final non-surgical interventions (step 3) (Additional file 1).

To date, it is unknown to what extent current Dutch OA care is consistent with the SCS, both with respect to the content of care as well as the sequence of care. Furthermore, there is a lack of clarity regarding the role of different health care providers in the performance of OA care according to the SCS. The SCS describes several interventions, but do not apportion these interventions to a specific discipline. It stands to reason that step-1 interventions mostly are performed by a GP. In case of unsatisfactory results, the GP refers patients to allied health care providers (step-2) or to an orthopaedic surgeon (step-3). However, the introduction of patient self-referral for physical therapy in 2006 [6], possibly has interrupted this natural sequence of care. It is expected that an increasing number of patients will leave out their GP and directly approach a physical therapist in case of experiencing musculoskeletal complaints [7]. In consequence, the question arises to what extent patients using self-referral for physical therapy still receive interventions described in step-1 of the SCS.

Therefore, the two main objectives of the present study are:

1. To describe the content of current GP care in patients with hip/knee OA, including the compliance to the SCS. 2. To describe the content of care in physical therapy practice in GP-referred versus self-referred patients.

## Methods

### Registration network

‘NIVEL Primary Care Database’ (NPCD) was used to achieve the research objectives [8]. This database contains data of several, separated primary care health care providers, including GPs and physical therapists (box 1). Participating GPs continuously record data on all patient contacts, including diagnoses, interventions, prescriptions and referrals [9]. GP-data are collected since 1992. For this study, data were used from 84 practices participating in NPCD. These practices provide a representative sample regarding gender and age in comparison with Dutch National Statistics.

**Table:** 

**Box 1** NIVEL Primary Care Database NIVEL Primary Care Database (In Dutch: NIVEL Zorgregistraties eerste lijn) uses routinely recorded data from health care providers to monitor health and utilisation of health services in a representative sample of the Dutch population. It includes data on health problems and treatment. The aim of NIVEL Primary Care Database is to monitor developments in health and the use of primary health services in the Netherlands. **Participants** of NIVEL Primary Care Database are: ▪ General practitioners ▪ Physical therapists ▪ Exercise therapists ▪ Dieticians ▪ Primary care psychologists ▪ GP out-of-hours services ▪ Health centres Gathered data are combined and supplemented with data of pharmaceutical care and secondary care collected by other organisations. **Privacy** NIVEL handles the data in accordance with the Dutch Data Protection Act. Researchers have no access to identifiable patient information, such as name, address or citizen service number. Research results cannot be traced back to individual persons, health care providers or health care organisations. Participating health care providers may withdraw from NIVEL Primary Care Database at any time, and without stating reasons. **Governance** Steering committees with representatives from national associations of health care providers decide about the use of the data.	

Participating physical therapists collect longitudinal data on patient characteristics, referrals, diagnoses, interventions and evaluations [10]. This part of the NPCD was constructed in 2001 and contains data of about 100 physical therapists, divided over 35 outpatient practices. The geographical distribution and the degree of urbanisation of the participating practices are in line with all Dutch physical therapy practices. In contrast to the representativeness of participating practices, participating physical therapists are more often male and are older compared to non-participating Dutch physical therapists.

### Participants

During 2006 to 2011, all patients with OA of the hip and/or knee who visited their GP and/or a physical therapist were selected from the NPCD. Hip/knee OA was operationalized by the ‘International Classification of Primary Care’ (ICPC) [11] codes L89 (hip OA) or L90 (knee OA). In physical therapy practice, in case of lacking ICPC codes, patients with hip/knee OA were identified by national diagnosis codes for allied health care, which are mandated by insurers.

### Medical record data

#### General practice

In general practice, first, patient characteristics (gender, age, location of OA (hip/knee/both hip and knee)) were collected. Second, GPs’ interventions were gathered, including (telephone) consults, home visits, prescriptions, and referrals. Prescriptions were registered according to the Anatomical Therapeutic Chemical (ATC) classification system [12]. With respect to the referrals, only referrals to physical therapists, dieticians, and orthopaedic surgeons were collected since these health care providers take part in the SCS.

#### Physical therapy

As in general practice, gender, age, and the location of OA of the participants were collected. Per treatment episode due to hip/knee OA, the applied interventions (information & advise/manual techniques/physical agent modalities/exercise therapy) and the amount of care (duration and number of sessions) were collected. Furthermore, to evaluate the effectiveness of the physical therapy episode, it was examined to what extent the formulated treatment goals were achieved (<25% / 25-50% / 50-75% / >75%) at the end of a treatment episode. Finally, the recurrence rate (recurrent complaint yes/no) and the type of access (referred by GP/referred by medical specialist/direct access) were gathered.

### Data analyses

Descriptive statistics were used to describe demographics of the OA population. The content of current care in general practice was described by considering the use of non-surgical treatment modalities proposed by the SCS. Operationalization of these treatment modalities in the NPCD was illustrated in Table 1. Due to the nature of the NPCD, several translations were necessary to enable interpretation of current registered OA care in terms of the SCS. Firstly, since education and lifestyle advises both were not registered in the NPCD, it was assumed that GPs educated their patients when ‘consults’ or ‘visits’ were registered in the medical record. Research by Noordman et al. showed that patients’ lifestyle is increasingly discussed during consultations in general practice, especially when it is relevant to patients’ complaints [13]. Secondly, in the NPCD, prescriptions and referrals were not necessarily directly linked to a specific diagnosis but to treatment episodes in which prescriptions or referrals were performed. Therefore, in case of prescriptions, we first selected the four most common drugs (4-digit ATC) which were applied especially to a diagnosis of hip/knee OA and subsequently counted the application of these prescriptions (NSAIDs, opioids, other analgesics and corticosteroids) in treatment episodes due to hip/knee OA. When appropriate, secondary analyses were performed to analyse the application of these prescriptions in more detail (7-digit ATC). Analyses of referrals occurred similarly; referrals to exercise therapy, dietary therapy and orthopaedic surgeons were selected.

**Table 1:** Operationalization of the content of current care in general practice in patients with hip/knee osteoarthritis

	*Treatment modality*	*Positively assessed if NIVEL Primary Care Database contains:*
Step 1 SCS	Education or lifestyle advise	≥1 consult or visit at the GP due to hip/knee OA
	Prescription of acetaminophen	≥1 prescription of other analgesics and antipyretics^a^
	Prescription of glucosaminesulphate	Not separately assessed but included in anti-inflammatory and anti-rheumatic products, non-steroids ^b^
Step 2 SCS	Prescription of (topical) NSAIDs	≥1 prescription anti-inflammatory and anti-rheumatic products, non-steroids ^b^
	Prescription of tramadol	≥1 prescription of opioids †
	Referral for exercise therapy	≥1 referral to physical therapy due to hip/knee OA
	Referral for dietary therapy	≥1 referral to dietary therapy due to hip/knee OA
Step 3 SCS	Referral to secondary care	≥1 referral to an orthopaedic surgeon due to hip/knee OA
	TENS	Not assessed
	Prescription intra-articular injections	≥1 Cyriax injection due to hip/knee OA
Remaining interventions	Prescription oral corticosteroid	≥1 prescription of corticosteroids for systemic use ‡ (without the application of a Cyriax injection)

Abbreviations: *OA* osteoarthritis, *SCS* Stepped-care strategy [21], *NSAID* non-steroidal anti-inflammatory drug

^a^Anatomical Therapeutic Chemical ((ATC) code N02B [12]

^b^ ATC code M01A

† ATC code N02A

‡ ATC code H02A

To determine the compliance of current GP care to the SCS, we assessed the proportion of patients who had been offered at least one treatment modality of step 1, respectively one or more treatment modalities of step-1 in addition to the application of a step-2 intervention, and the proportion of patients who used a step-1 and/or step-2 intervention in addition to a step-3 intervention.

To compare the content of care of self-referred patients and GP-referred patients visiting a physical therapist, two sample t-tests and chi-square tests were used, when appropriate. *P*-values of < .05 were considered statistically significant.

All statistical analyses were performed using Stata 12 (StataCorp LP, College Station, TX, USA) software.

## Results

### Patient characteristics

In total, 12118 patients with hip/knee OA were included from the NCPD; 11248 patients were extracted from general practice data and 870 patients were identified from physical therapy data. The majority of patients with hip/knee OA is female and suffers from knee OA. More patient characteristics are presented in Table 2.

**Table 2:** Characteristics of patients with hip/knee osteoarthritis in general practice and physical therapy practice (2006-2011)

	*General practice (n = 11248)*		*Physical therapy practice (n = 870)*	
Gender, female (n (%))	7552	(67)	581	(67)
Age, years (mean ± sd)	68.7 ± 12.4		66.7 ± 13.2	
Location of OA (n (%))				
Hip	4437	(39)	293	(34)
Knee	6462	(57)	577	(66)
Combination of hip and knee OA	349	(3)	Not applicable	

Abbreviations: *OA* osteoarthritis, *sd* standard deviation

### Content of GP care

**Fig. 1:**
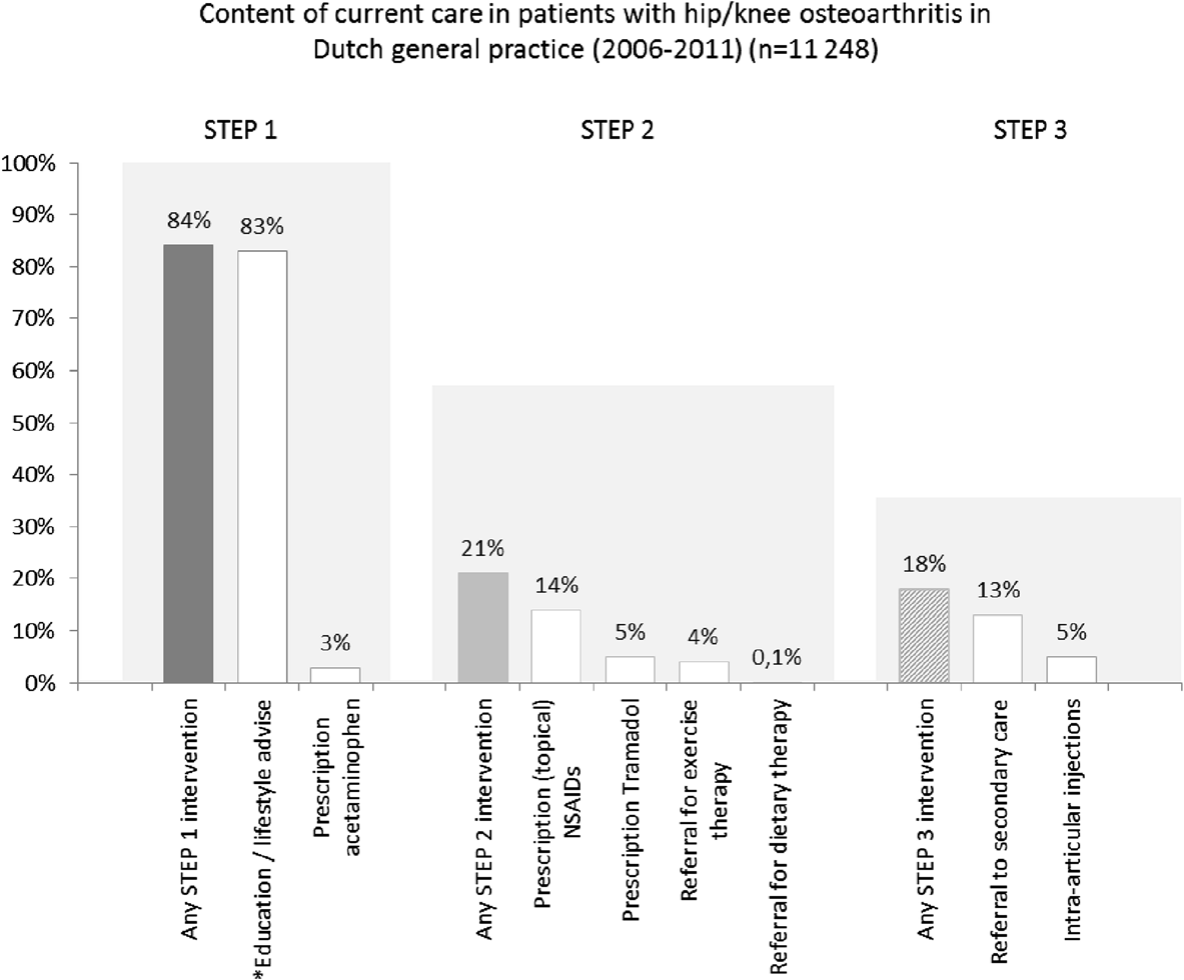
Content of current care in patients with hip/knee osteoarthritis in Dutch general practice (n = 11248). * Since education and lifestyle advises both were not registered in the NIVEL Primary Care Database, it was assumed that GPs educated their patients when ‘consults’ or ‘visits’ were registered in the medical record [14]

Figure 1 summarizes the content of current care in general practice, considering the different steps of the SCS. In total, 84% of the population was treated by at least one of the step-1 modalities, 21% was treated by any step-2 modality, and 18% received any step-3 intervention. Three percent of the patients received analgesics. NSAIDs were more frequently prescribed: more than two out of three patients treated by a step-2 intervention received NSAIDs. In 40% of the cases, the prescription of NSAIDs concerned Diclofenac or Diclophenac combinations. Ibuprofen, Meloxicam and Naproxen were prescribed in respectively 12%, 12% and 11% of the cases. In terms of numbers, referrals to orthopaedic surgeons were more often registered in the medical records than referrals to physical therapists (exercise therapy) and dieticians.

The extent to which GPs currently act in agreement with the SCS is shown in Table 3. It shows that 85% of the population who was treated by a step-2 modality also received any step-1 intervention. Seven percent exclusively received acetaminophen and six percent of the patients treated by a step-2 modality additionally received both step-1 interventions. Furthermore, in addition to the application of a step-3 intervention, 90% was treated by a step-1 modality as well. Twenty-seven percent of the step-3 population additionally received any step-2 intervention, mostly by NSAIDs (18%) and/or tramadol (7%). Two percent received both a prescription of NSAIDs or Tramadol and was referred to an allied health care provider. None of the patients receiving a step-3 intervention was offered all modalities described in step 1 and 2 (Table 3).

**Table 3:** Compliance to the Stepped-Care-Strategy in patients with hip/knee osteoarthritis in Dutch general practice (2006-2011) (n = 11 248)

		N	(%)
Step 1:	Number of patients who received ≥1 of the advised step-1 modalities	9396	(84)
	Education or lifestyle advise*	9332	(99)
	Prescription of acetaminophen	342	(4)
	Both modalities	278	(3)
Step 2:	Number of patients who received ≥1 of the advised step-2 modalities	2311	(21)
*Prior application of step-1 modalities:*	Any step 1 modality	1961	(85)
	1. Education or lifestyle advise*	1947	(85)
	2. Prescription of acetaminophen	153	(7)
	Both education (1.) & prescription (2.)	139	(6)
Step 3:	Number of patients who received ≥1 of the advised step-3 modalities	1988	(18)
*Prior application of step-1 modalities:*	Any step 1 modality	1794	(90)
	1. Education or lifestyle advise*	1791	(90)
	2. Prescription of acetaminophen	68	(3)
	Both education (1.) & prescription (2.)	65	(3)
*Prior application of step-2 modalities:*	Any step 2 modality	534	(27)
	3. Prescription of (topical) NSAID	365	(18)
	4. Prescription of tramadol	132	(7)
	5. Referral for physical therapy	143	(7)
	6. Referral for dietary therapy	5	(<1)
	Both prescription (3. or 4.) & referral (5. or 6.)	45	(2)
*Prior application of step-1 and step-2 modalities:*	Both education (1.) & prescription (2. & (3. or 4.)) & referral (5. or 6.)	0	(0)

Abbreviations: *n* Number, *NSAIDs* Non-Steroidal Anti-Inflammatory Drug

* Since education and lifestyle advises both were not registered in the NIVEL Primary Care Database, it was assumed that GPs educated their patients when ‘consults’ or ‘visits’ were registered in the medical record [13]

### Content of physical therapy

Almost two out of three of the patients suffering from hip/knee OA were referred by a GP (65%), 15% were referred by a medical specialist, and 20% of the patients with hip/knee OA visited a physical therapist on their own initiative. In a very small minority of patients (n = 10), physical therapists exclusively provided their patients information and advises. Due to this low number of patients, an extending evaluation of the implementation of (one element of) step-1 of the SCS in physical therapy practice was hindered. Although education rarely was applied as singular intervention, in 36% of the GP-referred population and 37% of the self-referred population, ‘information & advise’ was represented as a part of physical therapists’ treatment (*p* = .80) (Table 4).

**Table 4:** Treatment characteristics in patients with hip/knee osteoarthritis in Dutch physical therapy practice (2006-2011) (n = 870)

	*Total population (n = 870)*		*Referred by GP (n = 523)*		*Self-referred (n = 160)*		*p-value*
*Disease characteristics*							
Recurrent complaint, yes (n (%))^a^	297	(37)	171	(35)	70	(46)	*.01*
*Used interventions in ≥50% of the treatment sessions (n (%))* ^*b*^ *							
Information & advice	237	(37)	152	(36)	45	(37)	*.80*
Manual techniques	301	(47)	201	(58)	62	(51)	*.47*
Physical agent modalities	45	(7)	30	(7)	7	(6)	*.61*
Exercise therapy – functions	456	(72)	301	(71)	86	(71)	*.99*
Exercise therapy – activities	225	(35)	164	(39)	31	(26)	*<.01*
*Amount of care* ^c^							
Number of treatment sessions (mean ± sd)	10.0 ± 12.3		10.9 ± 13.5		8.6 ± 11.7		*.07*
Duration of treatment, weeks (mean ± sd)	9.1 ± 13.4		9.6 ± 12.8		9.0 ± 15.8		*.69*
Result* ^d^							
Treatment goals, ≥75% reached (n (%))	240	(71)	152	(72)	44	(70)	*.78*

Abbreviations: *GP* general practitioner, *sd* standard deviation

* Exclusively reported in finished treatment episodes (n = 788)

Number of missing values in total population: ^a^9%, ^b^20%, ^c^15%, ^d^57%

One difference between GP-referred and self-referred patients concerned the recurrence rate. Self-referred patients more often presented a recurrent complaint in comparison to GP-referred patients (*p* < .01). A trend was indicated with respect to less treatment sessions in the self-referred population compared to the GP-referred population. In both groups, exercise therapy was the most applied intervention, followed by manual techniques, and information & advise (Table 4). The focus of exercise therapy was not equal in both groups; although exercise therapy focussed on improving impairments of body functions was applied similarly in GP-referred and self-referred patients, GP-referred patients more often received exercise therapy focussed on improving limitations in activities compared to self-referred patients (*p* < .01).

## Discussion

In this study, we described the content of care registered in electronic records of 12.118 patients with hip/knee OA visiting their GP and/or physical therapist during 2006 to 2011, including the compliance to the SCS.

A remarkable result of our study comprised a lower prescription rate of pain medication (NSAIDs and acetaminophen) in patients with hip/knee OA in comparison to previous studies [14]; Belo J, Berger M, Koes B, Bierma Zeinstra SM (unpublished work). The lower use of acetaminophen and NSAIDs might be explained by the increasing availability of those (low-dosed) drugs over the counter. As a consequence, the total use of NSAIDs and analgesics in the OA population is probably underestimated in this study.

In line with the SCS, in Dutch general practice less advanced treatment modalities are generally more often applied than more advanced treatment modalities. However, only a small minority of patients is treated by a combination of different interventions belonging to one step before turning to the next step, within the time frame of our study. Most deviations from the SCS concern GPs’ prescriptions and their referral policy. With respect to GPs’ prescribed pain medication, our results show that NSAIDs (especially Diclophenac (combinations), Ibuprofen, Meloxicam and Naproxen) and tramadol (step-2 interventions) are more often prescribed than analgesics (step-1 intervention). This prescription behaviour has previously been indicated in an observational study by Cardol et al. [14]. Moreover, a more recent study investigating GPs’ attitudes regarding SCS recommendations, showed that 21% of the GPs (strongly) agree with the statement *‘NSAIDs should be the first choice of pain medication in patients with OA’* [15]. Given the recognized increased risk of several adverse outcomes in older adults due to the frequent use of NSAIDs and to improve guideline adherence, GPs could be advised to optimize the analgesics policy prior to consider NSAIDs prescription in patients with hip/knee OA [16]. Besides the prescription policy, deviations from the SCS are found regarding GPs’ referrals as well. In the NPCD, GPs registered fewer referrals to allied health care providers (exercise therapy, dietary therapy (step-2) than to orthopaedic surgeons (step-3). Partially, this could be explained by the moderate quality of the referral-registration in the medical records and the introduction of patient self-referral for allied health care. However, previous work, which has been published prior to the introduction of direct access of allied health care, also showed a lower referral rate for physical therapy compared to orthopaedic surgery [14]. Therefore, the question arises whether GPs could improve care by first ensuring optimal non-surgical care in primary care setting has been delivered, before referring to secondary care [17]. Fortunately, recent (unpublished) research in a population in which the SCS has been implemented showed that patients who are referred to secondary care are significantly more extensively treated by non-surgical interventions in primary care compared to patients who were not referred to secondary care (Barten JA, Smink AJ, Swinkels ICS, et al.)

The introduction of direct access to allied health care for example aimed to achieve a rearrangement of health care organization. Translated to OA care, it could have been expected that non-pharmacological step-1 interventions had been integrated in physical therapists’ treatment in case of patient self-referral. However, we did not indicate a difference with respect to the application of ‘information and advice’ between GP-referred and self-referred patients in physical therapy practice. Besides, only a handful of patients exclusively received education. The rearrangement of care, hence, seems to be in its infancy. It should be remarked that almost half of the patients using self-referral presented recurrent complaints (46%). These patients might have been treated by a step-1 intervention by a physical therapist or their GP, prior to the timeframe of this study. Further research is recommended to able an evaluation of the effects of task-shifting in OA-care.

As already mentioned, self-referred patients with hip/knee OA often present recurrent complaints in physical therapy practice. In accordance with studies in the general population and in patients with low back pain, the recurrence rate in self-referred patients significantly exceeds the recurrence rate in GP-referred patients [18, 19]. Patients with recurrent complaints might be more aware of direct accessibility and, therefore, are more likely to omit their GP in case of recognizable musculoskeletal complaints. This rationale is confirmed by research of Leemrijse et al. [18], indicating that the use of direct access was significantly higher in patients who received earlier treatment by a physical therapist.

Another difference between self-referred and GP-referred patients concerned the less frequent application of activities-related exercise therapy in self-referred patients. Commonly, treatment starts with improving impairments of body functions and gradually shifts to diminishing limitations in activities of daily life. At the same time, the role of the physical therapist changes from ‘hands-on therapist’ to ‘coach’ and the frequency of treatment sessions decreases. Possibly, this gradual phase out is less often used in patients who refers themselves. Physical therapists might focus on improving impairments, leaving the translation to activities of daily life to patients themselves. This situation stands to reason since a sizeable proportion of the self-referred patients has already gained some experience in the translation to daily life: recurrence rates are high. Furthermore, the lower amount of care in self-referred patients seems to support this rationale.

This study has some limitations. Firstly, in the NPCD, treatment episodes in general practice are constructed retrospectively. As a consequence, applied interventions (consults, prescriptions and referrals) were related to a treatment episode due to OA, unless they were aimed at treating any comorbidity. Secondly, both the increasing use of direct-access and the moderate registration of referrals in the medical record could have induced an underestimation of referrals to other health professionals, including physical therapy and dietary therapy. Since exercise therapy and encouraging weight loss are key recommendations in clinical guidelines for the treatment of lower limb OA [5], a higher referral rate than the indicated 5% respectively <1% could have been expected. Thirdly, we did not take into account the hierarchical structure of the data with patients nested in health professionals, nested in primary care practices both in general practice as well as in physical therapy practice. However, previous work showed that variances in health care use in patients with hip/knee OA were mainly located at patients’ level [20]. Fourthly, we were not able to evaluate thoroughly the sequence of the applied interventions in general practice, but evaluated which interventions from each step were applied in patients with hip/knee OA. Furthermore, we did not take into account whether a patient’s treatment was evaluated during an evaluation visit before turning to the next step, which is described as an integral part of the SCS [21]. Finally, data were extracted from two voluntary-based, separate registrations, both part of the NPCD. Selection bias could be excluded, as the number of patients objecting to participate in the NPCD is negligible and participating practices reflects the reality of Dutch general practices. As the NPCD comprises several, separate registrations, patients referred for physical therapy were not necessarily represented in the GP data and vice versa, disabling an evaluation of the compliance to the SCS in a singular patient by combining electronic data derived from several health professionals. At this moment, the NPCD is prepared to enable integration of data from several health professionals belonging to a singular patient. This opens the way to evaluate the compliance to the SCS more thoroughly, including the effects of using direct accessibility of allied health care on both patient-outcomes and the process of care.

## Conclusion

In accordance with the SCS, less advanced treatment modalities are more often applied in general practice than more advanced treatment modalities. However, completion of each SCS-step is achieved rarely. To optimize the adherence to the SCS, GPs could reconsider their analgesics policy prior to NSAID prescription and the low referral rate to exercise therapy and/or dietary therapy compared to orthopaedic surgeons. Self-referral to physical therapy partially distorts both the low referral rate in general practice and the low application rate of education as singular intervention in physical therapy practice. Compared to GP-referred patients, self-referred patients seems to be less intensively treated, possibly as a result of a more impairment-minded treatment strategy. This chosen strategy could be related to the higher recurrent rate in self-referred patients. Further research is recommended to evaluate more thoroughly the effects of task-shifting in OA care, taking into account the content and sequence of the SCS.

## Additional file

Additional file 1:
**Stepped-Care-Strategy ‘Beating OsteoARThritis’.**

## Abbreviations

OA: Osteoarthritis
SCS: Stepped-care-strategy
BART: Beating osteoARThritis
GP: General practitioner
NSAID: Non-steriodal anti-inflammatory drug
NPCD: NIVEL primary care database
ICPC: International classification of primary care
ATC: Anatomical therapeutic chemical
