# Supplementary material for: Treatment of hip/knee osteoarthritis in Dutch general practice and physical therapy practice: an observational study
Source: BMC Fam Pract. 2015 Jun 27;16:75. doi: 10.1186/s12875-015-0295-9 (PMC4483212; doi:10.1186/s12875-015-0295-9)
Supplement: Additional file 1: — Stepped-Care-Strategy ‘Beating OsteoARThritis’. [file 12875_2015_295_MOESM1_ESM.docx]

Additional_file_1:

**Stepped-Care-Strategy ‘Beating OsteoARThritis’[**[**6**](#_ENREF_6)**]**

|  | Step 1 | Step 2 | Step 3 |
| --- | --- | --- | --- |
| Diagnostic procedures and assessment | - Medical history and physical examination - Assessment function and activity limitations - Setting mutual goals | - Radiological assessment * - Assessment of pain coping and psychosocial factors - Adjust goals | - Consultation specialist - Adjust goals |
| Treatment modalities | Advised   - Education - Lifestyle advice - Acetaminophen | Advised   - Exercise therapy - Dietary therapy (in case of overweight) - (topical) NSAIDs or Tramadol |  |
|  | Optional:   - Glucosaminesulphate |  | Optional:   - Multidisciplinary care - TENS - Intra-articular injections |

Abbreviations: NSAIDs=Non-Steroidal Anti-Inflammatory Drugs, TENS=Transcutaneous Electrical Nerve Stimulation

* If there is a discrepancy between medical history and physical examination

† According to the definition overweight of the “Zorgstandaard obesitas NL” 2010: BMI>25kg/m2
